# Digital governance pressure and digital burnout among grassroots public sector employees: the mediating roles of cognitive load and negative affect

**DOI:** 10.3389/fpsyg.2026.1887098

**Published:** 2026-08-12

**Authors:** ZeKun Wang, Ting Wang, Hong Chen

**Affiliations:** 1 School of Public Administration and Law, Hunan Agricultural University, Changsha, China; 2 Research Center for Cultural Construction and Social Governance in Ethnic Regions, Guangxi Normal University, Guilin, China; 3 Yulin Normal University, Yulin, China

**Keywords:** cognitive load, digital burnout, grassroots public sector employees, negative affect, rural digital governance, SOR model

## Abstract

Digital technologies are reshaping public governance. Although they can improve administrative efficiency, service delivery, and governance capacity, they may also create occupational health risks for public sector employees. Existing research on digital burnout has largely focused on general technology-use settings, with limited attention to its formation in the public sector, especially among grassroots public sector employees. Drawing on the stimulus-organism-response (SOR) model and cognitive-affective theory, we examine how digital governance pressure is associated with digital burnout in rural digital governance in China. It develops a pressure stimulus-organism processing-burnout response framework to analyze the roles of technology-related, regulation-related, and context-related pressures and the mediating mechanisms of cognitive load and negative affect. Based on survey data from 535 grassroots public sector employees, the study applies partial least squares structural equation modeling (PLS-SEM) and necessary condition analysis (NCA) to examine both sufficient pathways and necessary-condition patterns. The PLS-SEM results show that technology-related pressures are associated with digital burnout through cognitive load and through the sequential pathway from cognitive load to negative affect. By contrast, regulation-related and context-related pressures are mainly associated with digital burnout through negative affect. The NCA results further suggest that, at high levels of digital burnout, the three forms of digital governance pressure, cognitive load, and negative affect exhibit threshold-like patterns, with cognitive load and negative affect showing relatively higher bottleneck values. These findings extend research on the unintended consequences of digital governance and provide empirical grounding for reducing digital burdens and protecting occupational health among grassroots public sector employees.

## 1 Introduction

Digital technology is reshaping public governance systems worldwide. It is widely viewed as a key instrument for improving administrative efficiency, optimizing public service delivery, and advancing governance modernization. In rural governance, digital technologies have changed both the form and content of governance work, accelerating a shift from manual, experience-based administration to data-, software-, and platform-based governance. However, as digital technology becomes increasingly embedded in everyday governance practice, accumulating evidence indicates that technological adoption does not automatically produce governance empowerment. The latent and negative functions of technology also require careful attention (Merton, 1936).

Digital technology can, for example, strengthen interaction between the public sector and society (Mergel et al., 2019). Yet these governance benefits may be uneven because of differences in regional development, digital infrastructure, and administrative capacity. Digital reform may therefore widen existing digital divides, a problem that is particularly salient in rural areas and in e-government practices in developing countries (Alfiani et al., 2024). Previous research has also noted that digital technology does not necessarily translate into effective governance. Although it reshapes governmental organizational structures, it may generate unintended effects such as mismatches between authority and responsibility, technological entanglement, and limited implementation capacity (Li, 2025).

For grassroots public sector employees, digital technology can improve work convenience and service efficiency. At the same time, tensions between technological rules and institutional logic may create digital administrative burdens (Fleischer and Wanckel, 2024; Peeters, 2023). These burdens may increase cognitive resource depletion and emotional strain among grassroots public sector employees (Islam et al., 2020; Li and Pang, 2026), thereby increasing the risk of digital burnout. Marienfeldt (2024) argues that digital tools are changing the work boundaries, discretionary space, and task structures of street-level bureaucrats, which may intensify work pressure and undermine occupational well-being. In rural China, the Digital Village Strategy has continued to advance nationwide. Grassroots public sector employees must not only learn and use various digital tools but also continuously translate between technological rules, institutional requirements, and real-world governance conditions (Ding, 2022; Dai et al., 2026). This makes them particularly vulnerable to digital burnout (Shu et al., 2025).

Digital burnout is an emerging occupational health problem in the digital era and reflects an unintended consequence of technology-enabled governance. Unlike traditional job burnout, digital burnout does not arise primarily from interpersonal interaction. Rather, it is shaped by dense digital connections and by patterns of interaction between individuals and digital technology systems (da Silva et al., 2024). This makes its accumulation less visible and potentially broader in scope than traditional job burnout (Erten and Özdemir, 2020; Zhao et al., 2026). Although digital burnout has attracted increasing scholarly attention, existing studies have focused mainly on the private sector and general occupational groups (Håkansta et al., 2025; Funke et al., 2026). Less is known about digital burnout in the public sector, especially among grassroots public sector employees. The public sector is both a driver of digital transformation and a key field in which digital technologies are applied. In practice, digital technologies have penetrated almost every stage of public governance (Wu, 2023). This is especially true for grassroots public sector employees, who must frequently use digital platforms and government software to perform governance tasks (Bovens and Zouridis, 2002) while bridging standardized digital procedures and differentiated grassroots realities (Ding, 2022). This dual pressure of technology use and institutional translation places them at a potentially high risk of digital burnout (Shu et al., 2025), making the issue worthy of further investigation.

Against this background, we examine the formation mechanism of digital burnout among grassroots public sector employees in rural digital governance in China. Building on the SOR model and the cognitive-affective dual-path perspective, it develops an analytical framework of pressure stimulus-organism processing-burnout response and examines how different forms of digital governance pressure are associated with digital burnout through cognitive and affective pathways. The study makes three contributions. First, it extends digital burnout research to rural digital governance in China by revealing the occupational health risks faced by grassroots public sector employees as implementers of digital governance. Second, based on the SOR model and the cognitive–affective perspective, this study analyzes the differentiated psychological mechanisms between digital governance pressure and digital burnout. The results show that different types of digital governance pressure are not associated with digital burnout in a homogeneous way. Instead, they operate through two differentiated psychological pathways: cognitive depletion and affective threat. Technology-related pressure is mainly transmitted through cognitive load and the sequential pathway of “cognitive load → negative affect.” In contrast, regulation-related pressure and context-related pressure are mainly associated with digital burnout through the pathway of negative affect. Third, by combining PLS-SEM and NCA, this study examines the formation of digital burnout from both sufficiency and necessity perspectives. This methodological combination helps identify sufficient pathways among digital governance pressure, cognitive load, negative affect, and digital burnout, while also clarifying the minimum conditions associated with high levels of digital burnout.

## 2 Literature review

### 2.1 The concept and manifestations of digital burnout

The theoretical roots of digital burnout can be traced to research on job burnout. In the 1970s, Freudenberger (1974) introduced the concept of job burnout and defined it as a state of physical and psychological exhaustion experienced under excessive work demands. Digital burnout can be understood as an extension of this concept in the digital era. As early as the late twentieth century, Brod (1984) noted that computer technology could produce negative psychological effects, including stress, anxiety, fatigue, and other burnout-related symptoms. Such problems may arise from continuous human-computer interaction, information-processing demands, and uncertainty in technology adaptation. With the widespread use of digital technologies, the Internet, and smart devices, digital burnout has gradually become an important research topic, although it remains insufficiently understood. Existing studies often define digital burnout as a fatigue and stress response caused by excessive use of digital devices (Erten and Özdemir, 2020; da Silva et al., 2024). It has been examined in several fields. In digital education, long-term online learning and platform-based teaching may lead to learning fatigue, reduced attention, and academic procrastination (Charoenporn et al., 2024). In healthcare, continuous use of electronic office systems and telemedicine platforms may increase information-processing burdens among medical staff and contribute to cognitive fatigue and emotional exhaustion (Asgari et al., 2024). In social media use, information overload, social pressure, and continuous screen exposure may trigger online withdrawal, digital disconnection, and social media burnout (Sheng et al., 2023). Although digital burnout manifests differently across fields, it generally involves cognitive, emotional, and behavioral dimensions. Erten and Özdemir (2020) identified three dimensions of digital burnout: emotional exhaustion, digital deprivation, and digital aging. Zhao et al. (2026) later expanded the dimensional structure to include digital aging, emotional exhaustion, cognitive overload, cognitive dissonance, digital deprivation, and behavioral addiction. These studies indicate that digital burnout is not a single fatigue response but a complex psychological state involving cognitive, emotional, and behavioral reactions. Based on this literature, the present study defines digital burnout among grassroots public sector employees as a comprehensive state of stress and exhaustion that emerges when employees use digital technology tools over long periods to perform governance tasks under the dual constraints of technological rules and bureaucratic institutional logic. This state includes cognitive fatigue, emotional exhaustion, and behavioral burnout. It differs from general technostress or short-term digital fatigue because it reflects a cumulative psychological condition formed through long-term digital work demands in public governance.

### 2.2 Conceptual distinctions between digital burnout and related concepts

Digital burnout is an emerging occupational health issue in the digital era. It is related to technostress, administrative burden, and occupational burnout in public sector occupational health research, but it differs from these concepts in important respects (Table 1).

**Table 1:** Conceptual distinctions between digital burnout and related concepts.

Concept	Technostress	Administrative burden	Occupational burnout	Digital burnout
Research perspective	Relationship between individuals and technology	Relationship between individuals and administrative systems	Relationship between individuals and work	Relationship between individuals and technology
Main focus	Users of digital technologies, information systems, or ICTs	Individuals interacting with administrative systems and policy procedures	Employees in the workplace	Individuals affected by digital technologies
Main features	Adaptive strain caused by technological characteristics in general settings, such as complexity and uncertainty	Increased costs experienced by individuals when interacting with administrative systems, such as learning costs, compliance costs, and psychological costs	A chronic syndrome caused by accumulated work stress, such as emotional exhaustion, depersonalization, and reduced performance	Negative individual reactions caused by the interaction between digital technology use and work or social contexts
Relationship with digital burnout	May serve as an antecedent of digital burnout	May serve as an antecedent of digital burnout	A broader concept and theoretical source of digital burnout	May be a specific form of occupational burnout; may also be an outcome of technostress and administrative burden

(1) Technostress and digital burnout: Research on technostress can be traced back to the 1980s. Brod (1984) defined technostress as a modern disease of adaptation caused by an inability to cope with new computer technologies in a healthy way. Technostress is not determined by technology alone; it is shaped by the interaction between the technological environment and users’ adaptive capacity (Caro and Sethi, 1985). It is therefore not merely a temporary difficulty in technology adaptation but a dynamic process triggered by stressors, perceived by individuals, and associated with multiple consequences in work and daily life (Califf and Brooks, 2020). Later studies conceptualized technostress as a multidimensional response driven by stressors such as techno-overload, techno-invasion, techno-complexity, techno-insecurity, and techno-uncertainty (Tarafdar et al., 2007).

The relationship between technostress and digital burnout has received increasing attention in recent years. In digital contexts such as social media use and healthcare services, information overload and intensive use of digital systems may increase cognitive load and further trigger fatigue, emotional exhaustion, and other burnout-related responses (Islam et al., 2020; Asgari et al., 2024). Some studies therefore regard technostress as an important antecedent of digital burnout and have confirmed its significant association with burnout-related outcomes (Wang et al., 2026). This literature suggests that technostress is closely related to digital burnout and can be viewed as one of its important antecedent conditions.

(2) Administrative burden and digital burnout: Research on administrative burden emerged around 2010, with intellectual roots in earlier work on red tape and pathological organizational rules. Early studies examined how excessive rules and complex procedures in public organizations affect the attitudes and behavior of civil servants (Bozeman, 1993). These studies showed that burdensome institutional requirements may reduce job satisfaction and organizational commitment while increasing stress, burnout, and other negative outcomes (George et al., 2021). Building on this literature, Burden et al. (2012) defined administrative burden as the burdensome experience perceived by individuals in policy implementation and public service interactions. Moynihan et al. (2015) further summarized administrative burden into learning costs, compliance costs, and psychological costs. In digital governance, digital technology is often expected to improve efficiency and reduce burdens. However, it may become intertwined with bureaucratic logic and generate new forms of digital administrative burden (Peeters, 2023). Some scholars therefore view digital burnout as a manifestation and institutional consequence of excessive administrative burden in digital governance contexts (Shu et al., 2025).

Administrative burden and digital burnout therefore emphasize different mechanisms. Administrative burden focuses on interactions between individuals and institutions and explains where institutional burdens originate. Digital burnout focuses on how such burdens accumulate into occupational health risks in digital environments.

(3) Occupational burnout and digital burnout: Occupational burnout is an important conceptual source of digital burnout. Traditional burnout research focuses on physical and psychological exhaustion that develops under long-term work stress, role conflict, emotional labor, and insufficient resources (Maslach and Jackson, 1981). The World Health Organization defines burnout as an occupational phenomenon resulting from chronic workplace stress that has not been successfully managed; its main features include energy depletion, depersonalization, and reduced professional efficacy (World Health Organization, 2019). As digital technology has become increasingly embedded in work organizations and social life, the concept of digital burnout has entered academic discussion. Scholars argue that digital burnout is a specific occupational health problem in the digital era and can be viewed as an extension of traditional occupational burnout in digital contexts (Li and Pang, 2026). Compared with occupational burnout, digital burnout places greater emphasis on the continuous depletion of cognitive and emotional resources caused by digitalized environments (Zhao et al., 2026).

Collectively, technostress, administrative burden, and occupational burnout provide important theoretical foundations for understanding digital burnout, but digital burnout is not a simple substitute for any of these concepts. Technostress focuses on interactions between individuals and technology and explains how technology creates stress. Administrative burden focuses on interactions between individuals and institutions and explains how institutional burdens arise. Occupational burnout focuses on interactions between individuals and work and explains how long-term work-related depletion leads to physical and psychological exhaustion. Within our analytical context, digital burnout does not arise from technological, institutional, or occupational factors alone. Instead, it emerges from their interaction. It inherits the core logic of occupational burnout, incorporates the concern of technostress with human-technology adaptive strain, and draws on administrative burden theory to explain institutional compliance costs. Digital burnout can therefore be understood as a more inclusive and context-specific concept in the digital era.

### 2.3 Digital burnout among grassroots public sector employees

With the deep integration of digital technologies into public governance, digital burnout is no longer confined to general digital contexts. Public sector employees also face digital burnout risks, yet existing studies have paid limited attention to burnout among grassroots public sector employees in digital governance. Related studies generally explain the effects of digital empowerment, technostress, and administrative burden from three perspectives: technological, institutional, and actor oriented. The technological perspective emphasizes how digital tools reshape work processes and increase technology-related demands. Studies have shown that the use of digital platforms, algorithmic systems, and government software may increase techno-overload, techno-complexity, and techno-uncertainty, thereby weakening employees’ psychological well-being and work enthusiasm (Bondanini et al., 2020; Ayyagari et al., 2011). A study on technostress and employee well-being found a close relationship between technostress and employees’ mental health. Techno-overload and techno-invasion were among the most frequently reported negative stressors, and they were associated with adverse outcomes such as cognitive overload and emotional exhaustion (Mansuroğlu and Smith, 2026). Another study on the technostress-employee mental health link found that technostress may increase employee burnout (Funke et al., 2026). These studies show that the technological perspective is useful for analyzing the backlash effects of digital governance and suggests that the expansion and embedding of digital technology can create psychological pressure among public sector employees (Consiglio et al., 2023).

The institutional perspective usually builds on administrative burden theory to analyze the backlash effects of digital governance. It suggests that the extension and embedding of bureaucratic logic into digital spaces may generate new institutional pressures and administrative burdens (Shu et al., 2025; Liu et al., 2025), which may further increase the workload and psychological burden of grassroots public sector employees. Studies have shown that the embedding of digital technology in bureaucratic organizations may lead to the digital distortion of red tape. Public officials are required to learn and follow digital application procedures, online operating rules, and specific data formats (Heggertveit et al., 2022). This process may increase learning costs, compliance costs, and psychological costs. It may also create new forms of digital administrative burden (Peeters, 2023).

The actor-oriented perspective is grounded in street-level bureaucracy theory (Lipsky, 1980). It suggests that the embedding of digital technologies may move grassroots public sector employees toward an implementation mode centered on algorithms, systems, and screens (Bovens and Zouridis, 2002). This process may compress their discretionary space (Buffat, 2015; Busch and Zinner Henriksen, 2018) and reduce their professional agency. Some scholars argue that digital technology is reshaping the work patterns of street-level bureaucrats. Their work structures and action spaces are increasingly constrained by information systems and algorithmic rules (Marienfeldt, 2024). At the same time, digital channels may lower the threshold for public expression and public complaints. As a result, street-level bureaucrats are more frequently exposed to online complaints and immediate accountability pressure (Zhao et al., 2026). This continuous external exposure pressure interacts with the compression of internal discretionary space and may increase occupational stress.

These studies provide an important basis for understanding digital burnout among grassroots public sector employees by explaining the effects of digital technologies from technological, institutional, and actor-oriented perspectives. However, most studies remain limited to single theoretical perspectives. They have not yet provided a systematic explanation of how digital technology rules, bureaucratic institutional logic, and complex grassroots governance contexts jointly generate psychological pressure. To bridge this gap, we integrate technological, institutional, and actor-oriented perspectives and draws on the SOR model and cognitive-affective theory. It conceptualizes digital governance pressure as three forms of external stimuli: technology-related pressure, regulation-related pressure, and context-related pressure. Cognitive load and negative affect are treated as organismic states, whereas digital burnout is regarded as the response. On this basis, the study constructs a stimulus-organism-response model of digital burnout among grassroots public sector employees.

## 3 Theoretical model and research hypotheses

### 3.1 Theoretical analytical framework

The SOR model was originally proposed by Mehrabian and Russell (1974) to explain how environmental characteristics influence individuals’ psychological states and ultimately shape behavioral responses. The model suggests that human behavior is not a mechanical reaction to external stimuli; instead, it results from individuals’ perception of environmental stimuli and subsequent internal psychological processing (Mehrabian and Russell, 1974). In the SOR model, stimulus refers to antecedent conditions, organism refers to the internal mediating state, and response refers to the outcome. The SOR model has been widely applied across disciplines, including information systems, behavioral psychology, and public administration, and has become a useful framework for analyzing individual behavior and psychological decision-making because of its strong contextual adaptability and explanatory power (Bigné et al., 2020; Hwang et al., 2020). Many scholars have used this model to examine how individuals respond to technological disturbance or organizational pressure. For example, Zhang et al. (2025) used the SOR model to examine the relationship between online office application use and employees’ psychological fatigue. Cao et al. (2020) also showed that the SOR model can explain how external digital stimuli influence subsequent behavioral responses through individuals’ internal states. The SOR model therefore provides a suitable theoretical framework for analyzing how digital governance pressure is associated with digital burnout among grassroots public sector employees.

In rural digital governance in China, the external stimuli faced by grassroots public sector employees are not limited to a single form of technostress. They instead constitute a complex set of digital governance pressures shaped by technological rules, institutional logic, and governance contexts (Wang et al., 2024). In practice, grassroots public sector employees must learn and operate multiple systems, software programs, and digital platforms. They must also follow institutional procedures to complete data reporting and maintain digital records of governance activities. At the same time, they need to translate and adapt governance tasks between digital rules and grassroots realities. Building on this understanding, we integrate technological, institutional, and actor-oriented perspectives and conceptualizes the external stimuli perceived by grassroots public sector employees in digital governance practice as three types of pressure: technology-related pressure, regulation-related pressure, and context-related pressure. Technology-related pressure mainly arises from learning platform operations, adapting to system functions, and coping with continuous updates of digital platforms and government systems (Tarafdar et al., 2007; Kumar, 2024). Regulation-related pressure mainly stems from rule constraints reinforced by institutional requirements and performance evaluation (Moynihan et al., 2015; Liu et al., 2025). It mainly reflects vertical pressure from higher-level organizations, institutional rules, and accountability chains. This includes pressure related to online reporting, format requirements, digital trace-making, performance evaluation, and accountability (Heggertveit et al., 2022; Zhao et al., 2026). Context-related pressure arises from the tension between standardized digital rules and differentiated grassroots realities. It is reflected in the additional pressure of explanation, translation, and coordination experienced by grassroots public sector employees when they face non-standardized tasks in grassroots governance and complex situations that are difficult for digital systems to fully cover (Dai et al., 2026). This study treats these three types of pressure as stimulus variables in the SOR model.

The organism refers to the internal psychological state generated under the influence of external stimuli. To specify this internal response, we introduce cognitive-affective theory as a complementary explanation. The theory suggests that individuals’ responses to stressors are shaped by two mediating pathways: cognitive appraisal and affective arousal (Lazarus and Folkman, 1984). In public administration, studies show that public sector employees do not directly develop burnout when facing role stress, emotional demands, or administrative burdens. Instead, burnout-related outcomes are formed through mechanisms such as stress appraisal, psychological capital, emotional labor, and organizational affective support (Eldor, 2018; Grandey and Gabriel, 2015). These mechanisms may further manifest as emotional exhaustion, psychological fatigue, and behavioral burnout (Fuenzalida et al., 2024). For example, Afzal and Panagiotopoulos (2024) argue that, in increasingly complex digital work environments, information overload and frequent external stimuli do not directly cause withdrawal behavior among frontline public officials. Instead, they first trigger perceptions of cognitive overload, which then activate negative affect, such as technology anxiety and irritation. Following this logic, we define cognitive load and negative affect as organism-level psychological responses of grassroots public sector employees under external digital governance pressure in rural China.

**Figure 1:**
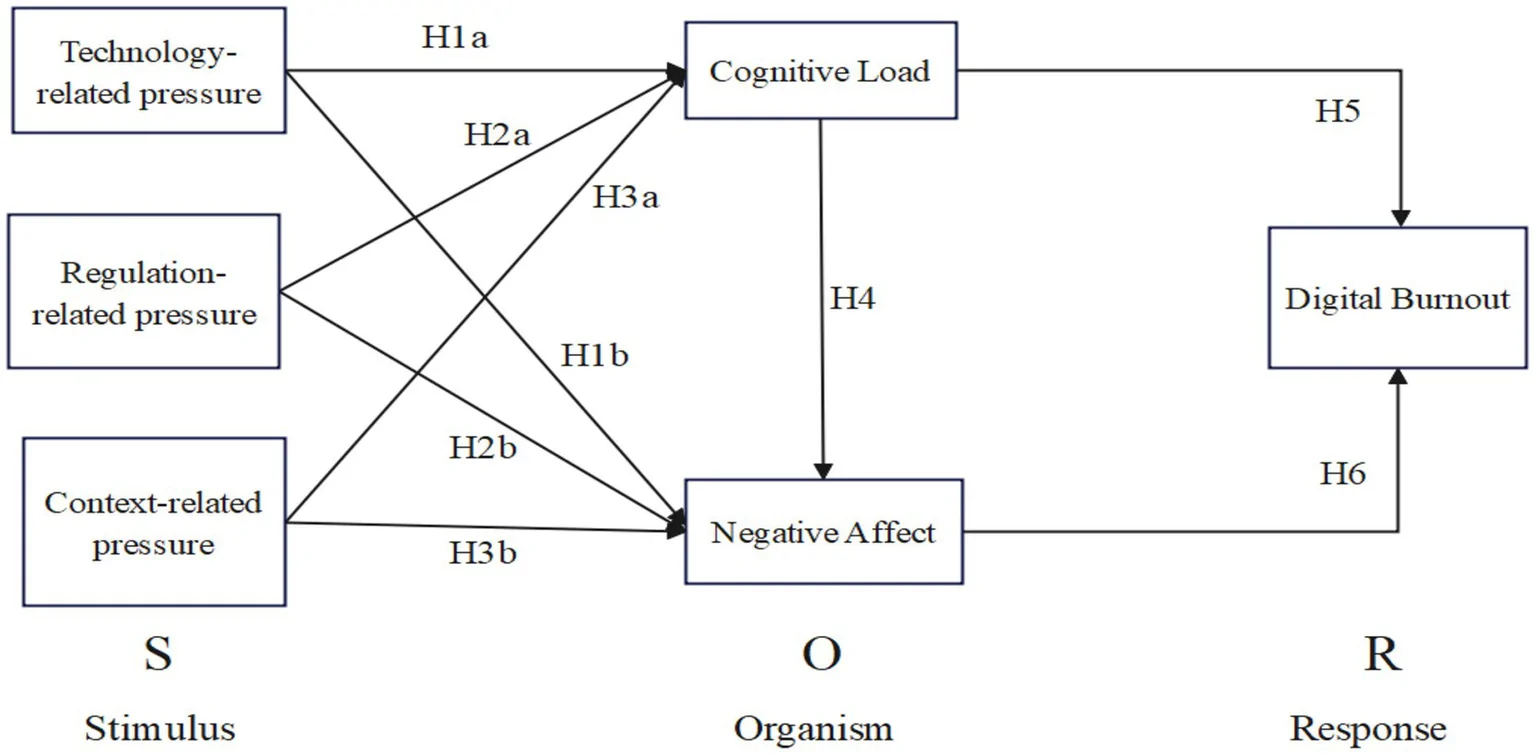
The theoretical framework of this study.

Response refers to the final outcome that emerges after individuals experience external stimuli and internal psychological processing. Within our framework, digital burnout is regarded as a psychological outcome formed after grassroots public sector employees are exposed to digital governance pressure over a long period. As defined above, digital burnout refers to cognitive fatigue, emotional exhaustion, and behavioral burnout that emerge when grassroots public sector employees continuously use digital technology tools to perform governance work and gradually deplete their cognitive and emotional resources (Erten and Özdemir, 2020). This state is not a direct reflection of external digital pressure. Rather, it results from the cumulative effects of cognitive load and negative affect. Therefore, we treat digital burnout as the final response variable in the SOR model (see Figure 1).

### 3.2 Research hypotheses

#### 3.2.1 Technology-related pressure, cognitive load, and negative affect

Technology-related pressure refers to the technological burden and adaptation pressure experienced by grassroots public sector employees during digital governance (Consiglio et al., 2023; Bondanini et al., 2020). It arises from complex technological rules, frequent system updates, and changing operational requirements. In China’s Digital Village Strategy, digital technologies have not simply replaced traditional governance tasks. Instead, they have created a dual-track work pattern in which offline governance and online documentation operate in parallel (Ding, 2022; Wu, 2023). On the one hand, grassroots public sector employees must maintain traditional place-based governance responsibilities, such as face-to-face public service, conflict mediation, and policy implementation. On the other hand, they must continuously learn and adapt to changing digital systems and platform rules to complete routine digital trace-making tasks, such as data reporting and activity log submission. Under these conditions, digital technology is no longer merely an auxiliary tool; it has become a technological environment that grassroots public sector employees must continuously learn, understand, and adapt to (Jørring et al., 2025). From a cognitive perspective, individuals have limited cognitive resources (Sweller, 1988). When grassroots public sector employees work in an environment characterized by multiple platforms, different rules, and frequently changing operational standards, their attention allocation and information-processing burden increase. This may lead to a higher level of cognitive load. From an affective perspective, continuous technology learning and uncertainty in system operation may trigger negative affect, including technology anxiety, doubts about self-efficacy, and helplessness (Kumar, 2024). Taken together, these arguments lead to the following hypotheses:

*H1a*: Technology-related pressure is positively associated with cognitive load among grassroots public sector employees.

*H1b*: Technology-related pressure is positively associated with negative affect among grassroots public sector employees.

#### 3.2.2 Regulation-related pressure, cognitive load, and negative affect

Regulation-related pressure refers to governance pressure experienced by grassroots public sector employees during digital governance because of institutional requirements and performance accountability. The perspective of technology-organization mutual shaping suggests that technology is not adopted in a fixed form; rather, it is shaped by existing institutional rules and becomes technology-in-practice (Fountain, 2001). When the connective features of digital technology are absorbed by the rigid accountability logic of bureaucracy, unintended paradoxes of technological governance may emerge. Tools originally designed to empower officials and reduce burdens may be transformed into instruments of monitoring and accountability (Wang et al., 2024). This transformation reduces tolerance for error in grassroots governance and adds the psychological cost of uncertain sanctions to traditional compliance costs. Under this logic, grassroots public sector employees must follow a rigid rule of getting things done, following rules, and leaving digital traces (Ren et al., 2026; Sun and Cong, 2024). From a cognitive perspective, regulation-related pressure requires employees to pay close attention to data formats, procedural standards, and the completeness of digital records to avoid rework or accountability caused by mistakes. It may therefore increase the burden of rule identification and information verification (Moynihan et al., 2015; Peeters, 2023). From an affective perspective, when operational mistakes are no longer merely technical errors but may lead to public criticism, score deductions, or performance sanctions, regulation-related pressure becomes internalized as persistent psychological tension and accountability anxiety (Hattke et al., 2020; Overman et al., 2025). Thus, regulation-related pressure has a dual nature: it involves both cognitive processing costs and emotional threat effects (Mitchell et al., 2018; Jang and Kim, 2021; Liu et al., 2025). The above reasoning suggests the following hypotheses:

*H2a*: Regulation-related pressure is positively associated with cognitive load among grassroots public sector employees.

*H2b*: Regulation-related pressure is positively associated with negative affect among grassroots public sector employees.

#### 3.2.3 Context-related pressure, cognitive load, and negative affect

Context-related pressure refers to the pressure experienced by grassroots public sector employees when they must interpret, adjust, and contextualize digital governance rules in public governance. This pressure arises from the tension between digital governance rules and complex grassroots governance realities. Unlike technology-related and regulation-related pressure, context-related pressure does not originate directly from technical systems or institutional rules themselves. Instead, it stems from the mismatch between these systems and rules and the rural governance context. In rural digital governance practice, government departments often transform complex, diverse, and context-specific governance processes into quantifiable data and standardized procedures to enable process supervision, performance evaluation, and responsibility tracking (Bovens and Zouridis, 2002). However, grassroots governance often involves highly contextualized and non-standardized problems. Many tasks cannot be fully carried out according to the rule frameworks preset by digital systems (Dai et al., 2026). When digital technology rules, bureaucratic institutional logic, and complex governance contexts do not fully align, grassroots public sector employees often need to interpret, communicate, and coordinate among them to complete governance tasks. This process may increase the burden of information understanding, contextual judgment, and communication coordination (Li and Pang, 2026). It may also generate negative affect, such as anxiety, helplessness, and frustration, because of implementation obstacles, repeated communication, and a reduced sense of governance efficacy (Herd et al., 2023). Accordingly, we advance the following hypotheses:

*H3a*: Context-related pressure is positively associated with cognitive load among grassroots public sector employees.

*H3b*: Context-related pressure is positively associated with negative affect among grassroots public sector employees.

#### 3.2.4 Cognitive load and negative affect

Cognitive load refers to the burden placed on individuals’ mental resources during information processing and task performance. According to cognitive-affective theory, external stimuli do not directly trigger affective responses. Instead, individuals first appraise these stimuli cognitively, and this appraisal then activates corresponding affective experiences (Sweller, 1988; Paas et al., 2003). When task demands continuously exceed individuals’ cognitive processing capacity, they may experience not only exhaustion in judgment and task execution but also negative affect such as tension and anxiety. In rural digital governance in China, cognitive load among grassroots public sector employees is not an occasional fluctuation. it is better understood as a persistent form of psychological depletion caused by multitasking and the long-term accumulation of cognitive overload (Håkansta et al., 2025; Jørring et al., 2025). A prolonged state of high cognitive load continuously consumes attention and emotional regulation resources, which may further intensify negative affect. Cognitive load is therefore not only a key mediator in the transmission of external pressure but also an important antecedent of negative affect. From this, we derive the following hypothesis:

*H4*: Cognitive load is positively associated with negative affect among grassroots public sector employees.

#### 3.2.5 Cognitive load, negative affect, and digital burnout

As discussed above, digital burnout is not an immediate response to external digital governance pressure it instead gradually accumulates through the depletion of cognitive and affective resources in a continuous digital work context. From a cognitive perspective, when grassroots public sector employees remain under high cognitive load for a long period, their limited attention, memory, and information-processing resources are continuously consumed. This may lead to attention deficits and cognitive fatigue (Koutsimani et al., 2021). As cognitive resources are depleted, employees are more likely to feel exhausted and burned out when performing digital governance tasks. From an affective perspective, repeated experiences of negative affect, such as tension, anxiety, and helplessness, may gradually drain emotional resources. Over time, this may lead to emotional exhaustion, psychological resistance, and behavioral burnout (Peng and Li, 2023). Building on the above, we propose:

*H5*: Cognitive load is positively associated with digital burnout among grassroots public sector employees.

*H6*: Negative affect is positively associated with digital burnout among grassroots public sector employees.

#### 3.2.6 The mediating mechanisms of cognitive load and negative affect

According to the SOR model, external stimuli are not directly transformed into psychological outcomes. Instead, this transformation occurs through internal processing within the organism (Mehrabian and Russell, 1974). In rural digital governance in China, technology-related pressure, regulation-related pressure, and context-related pressure do not operate in isolation. They are instead associated with digital burnout through the combined mechanisms of cognitive load and negative affect. Cognitive load and negative affect therefore serve as key psychological mechanisms linking external pressure to digital burnout. In particular, technology-related, regulation-related, and context-related pressure may increase cognitive load among grassroots public sector employees and may also trigger negative affect, such as tension, anxiety, and unease. At the same time, cognitive load may further intensify negative affect. Together, cognitive load and negative affect contribute to the formation of digital burnout. These considerations motivate the following hypotheses:

*H7a*: Cognitive load mediates the relationship between technology-related pressure and digital burnout.

*H7b*: Cognitive load mediates the relationship between regulation-related pressure and digital burnout.

*H7c*: Cognitive load mediates the relationship between context-related pressure and digital burnout.

*H8a*: Negative affect mediates the relationship between technology-related pressure and digital burnout.

*H8b*: Negative affect mediates the relationship between regulation-related pressure and digital burnout.

*H8c*: Negative affect mediates the relationship between context-related pressure and digital burnout.

*H9a*: Cognitive load and negative affect play a chain-mediating role in the relationship between technology-related pressure and digital burnout.

*H9b*: Cognitive load and negative affect play a chain-mediating role in the relationship between regulation-related pressure and digital burnout.

*H9c*: Cognitive load and negative affect play a chain-mediating role in the relationship between context-related pressure and digital burnout.

## 4 Research design

### 4.1 Variable measurement

The independent variables in our research are technology-related pressure, regulation-related pressure, and context-related pressure. Technology-related pressure refers to the technological burden experienced by grassroots public sector employees because of complex digital system operations, frequent system updates, and high learning requirements. Its measurement draws mainly on studies of technostress creators, technological complexity, technological uncertainty, and technology adaptation by Tarafdar et al. (2007), Ragu-Nathan et al. (2008), and Ayyagari et al. (2011). The items were adapted to the context of grassroots digital governance. Regulation-related pressure mainly arises from rule constraints strengthened by institutional requirements and performance evaluation. Its measurement builds on Moynihan et al. (2015) and Liu et al. (2025), who examined administrative burden and digital red tape, and on Mitchell et al. (2018) and Jang and Kim (2021), who discussed performance evaluation, supervisory control, and outcome assessment. Based on these studies, the items were adapted to capture online reporting, format requirements, digital trace-making, and accountability pressure in grassroots digital governance. Context-related pressure refers to the pressure experienced when grassroots public sector employees must interpret, adjust, and contextualize digital governance rules in public governance. It arises from the tension between digital governance rules and complex grassroots governance realities. Its measurement draws mainly on Lipsky (1980), Bovens and Zouridis (2002), and Busch and Zinner Henriksen (2018), who discussed task-technology fit, street-level bureaucracy, system-level bureaucracy, and digital discretion. The items were adapted to the Chinese grassroots digital governance context and capture the additional pressure of explanation, communication, and coordination when employees deal with non-standardized tasks and complex situations that digital systems cannot fully cover.

The mediating variables are cognitive load and negative affect. Cognitive load refers to the information-processing burden, attention allocation, and mental effort perceived by grassroots public sector employees when performing digital governance tasks. Its measurement draws mainly on Sweller (1988), Paas et al. (2003), and Leppink et al. (2013). Negative affect refers to negative emotional experiences in digital governance work, such as tension, anxiety, and helplessness. Its measurement draws mainly on the negative affect dimension of the PANAS scale developed by Watson et al. (1988). The items were adapted to fit the context of grassroots digital governance work.

Digital burnout is the dependent variable. It refers to cognitive fatigue, emotional exhaustion, and behavioral burnout formed among grassroots public sector employees during long-term digital governance work. Its measurement adapts the classic burnout scale developed by Maslach and Jackson (1981) and on digital burnout scales developed by Erten and Özdemir (2020) and Zhao et al. (2026). The items were revised to fit the context of grassroots digital governance in China.

All latent variables were measured using a five-point Likert scale ranging from 1 (strongly disagree) to 5 (strongly agree).

To ensure content validity and contextual fit, we first developed an initial pool of measurement items based on the literature review and contextual adaptation. Two scholars in public management and one scholar in applied psychology were then invited to review the content validity of the items. They assessed whether the items accurately reflected the conceptual domain of each construct, whether the wording was clear and unambiguous, and whether the items were consistent with the context of grassroots digital governance. Based on their feedback, items with overlapping meanings, weak contextual relevance, or potential ambiguity were removed, and several items were revised to better fit the research context. To further improve contextual fit and content validity, a small-scale pilot test was conducted in Guiyang, China, before the formal survey (n = 19). The pilot participants were grassroots public sector employees with experience using government digital platforms or grassroots digital governance tools. The pilot test focused on whether the items were easy to understand, matched the work experience of grassroots digital governance, and avoided ambiguous wording or repeated meanings. Based on the pilot feedback, some item wordings were further refined to reflect the actual work context of grassroots public sector employees. The final measurement items are shown in Table 2.

**Table 2:** Conceptualization of constructs and measurement items.

Variable	Item	Construct	References
Technology-related pressure	TRP1	The digital platforms and government systems I use at work have relatively complex operating rules.	Tarafdar et al. (2007), Ragu-Nathan et al. (2008), Ayyagari et al. (2011)
	TRP2	The digital systems or government software I use at work are updated relatively frequently.	
	TRP3	Using digital platforms and government systems usually requires a certain level of digital skills.	
	TRP4	The functional design of some digital platforms or government systems does not fully match actual work needs.	
Regulation-related pressure	RRP1	Platforms and systems impose very strict requirements on data formats and reporting standards.	Liu et al. (2025), Moynihan et al. (2015)
	RRP2	The requirements for data definitions and reporting rules are not fully consistent across platforms used by different departments	
	RRP3	The completeness of online documentation is often used to evaluate work completion.	
	RRP4	Rankings, notifications, or feedback results on digital platforms are often linked to performance evaluation.	
Context-related pressure	CRP1	When implementing digital tasks, I often need to explain online procedures and specific requirements to villagers.	Lipsky (1980), Bovens and Zouridis (2002), Busch and Zinner Henriksen (2018)
	CRP2	When villagers are unfamiliar with digital platform operations, I often need to help them complete the relevant online procedures.	
	CRP3	When digital governance tasks are implemented in rural areas, their execution often needs to be adjusted according to local conditions.	
	CRP4	When platform procedures are inconsistent with villagers’ actual needs, I often need to communicate and coordinate with them.	
Cognitive Load	CL1	When completing digital governance tasks, I need to invest substantial mental effort in thinking, judgment, and memory.	Sweller (1988), Paas et al. (2003)
	CL2	The amount of information involved in digital governance work exceeds what I can effectively process.	
	CL3	Repeatedly switching between online and offline work increases my mental burden.	
	CL4	The operational and reporting requirements on digital platforms take up a large amount of my attention and mental resources.	
Negative Affect	NA1	The continuous task requirements in digital governance work easily make me feel tense.	Watson et al. (1988), Pircher Verdorfer (2024)
	NA2	The online processing requirements in digital governance work easily make me feel anxious.	
	NA3	The continuous handling of digital platform-related matters easily makes me feel irritated.	
	NA4	The increasing demands of digital work often make me feel helpless.	
Digital Burnout	DB1	Long-term engagement in digital work has left me physically and mentally exhausted.	Maslach and Jackson (1981), Erten and Özdemir (2020), Zhao et al. (2026)
	DB2	I have gradually developed a sense of weariness towards digital work tasks.	
	DB3	When facing digital platforms and systems, I often feel low in energy.	
	DB4	I sometimes reduce my active engagement in digital work tasks.	

### 4.2 Data sources

This study collected data mainly through an online questionnaire survey. The respondents were grassroots public sector employees directly involved in rural digital governance, including village cadres, township public institution staff, and township civil servants. Given the geographically dispersed distribution of grassroots public sector employees in rural areas, we employed a combination of convenience sampling and snowball sampling. Shanghai, Hangzhou, Wuhan, Changsha, Guiyang, and Chengdu were selected as survey cities from eastern, central, and western China. These cities were selected to increase regional heterogeneity rather than to construct a nationally representative probability sample. Shanghai and Hangzhou represent eastern China, where digital governance infrastructure is relatively well developed. Wuhan and Changsha represent central China, where digital governance practices are active. Guiyang and Chengdu represent western China, where rural digital governance has also been actively promoted.

With the support of government departments, the research team relied on grassroots contact channels established during previous fieldwork and distributed the questionnaire to work-related WeChat groups in different regions to obtain the initial sample. Initial respondents were then invited to forward the questionnaire to work groups across villages and townships, thereby expanding sample coverage. To ensure sample relevance, respondents had to be grassroots public sector employees who regularly used digital governance platforms, government information systems, or other digital tools in their daily work. Respondents without such experience, those with incomplete responses, and those who failed quality checks were excluded from the final sample.

Procedural controls were applied during questionnaire design, distribution, and data cleaning. These controls included explaining the research purpose to respondents, ensuring anonymity and voluntary participation, using neutral wording in the questionnaire items, and requiring respondents to have practical work experience with government digital platforms or tools. A total of 621 questionnaires were distributed. After invalid responses with very short completion times or missing key items were excluded, 535 valid questionnaires remained. The basic characteristics of the sample are shown in Table 3.

**Table 3:** Sample characteristics.

Item	Category	Frequency	Percentage (%)
Gender	Male	337	62.99
	Female	198	37.01
Age	25 years of age or younger	64	11.96
	26–35 years old	273	51.03
	36–45 years old	137	25.61
	46 years of age and above	61	11.4
Highest education	High school and below	32	5.98
	Junior college	81	15.14
	Bachelor’s degree	312	58.32
	Master’s degree or above	110	20.56
Years of rural work experience	1–3 years	134	25.05
	4–6 years	189	35.33
	7–10 years	121	22.62
	More than 10 years	91	17.01
Job Type	Village cadres	180	33.64
	Staff of township public institutions	269	50.28
	Township civil servants	86	16.07

*N* = 535. Percentages may not sum to 100 due to rounding.

### 4.3 Research methods

This study used a combined approach of partial least squares structural equation modeling (PLS-SEM) and necessary condition analysis (NCA). PLS-SEM was selected for two main reasons. First, the study aims to explain and predict the formation mechanism of digital burnout among grassroots public sector employees. The focus is not only on overall model fit but also on identifying the pathways through which different types of digital governance pressure are associated with digital burnout. Second, several constructs were adapted from existing scales to the context of rural digital governance in China, making PLS-SEM suitable for testing an exploratory and prediction-oriented model. NCA was used as a complementary analysis because PLS-SEM and NCA follow different causal logics. PLS-SEM is mainly based on sufficiency logic and examines whether an antecedent variable has a significant effect on an outcome variable. Put differently, it identifies which factors are associated with the formation of digital burnout. By contrast, NCA is based on necessity logic and can determine whether a given condition must reach a certain level for the outcome to reach a high level. In the present analysis, NCA is used to examine which conditions are indispensable when high levels of digital burnout are observed. Combining PLS-SEM and NCA therefore helps reveal the formation mechanism of digital burnout among grassroots public sector employees from both sufficient-pathway and necessary-condition perspectives.

The study used SPSS 26.0, SmartPLS 4.0, and the built-in NCA module in SmartPLS to analyze the sample data. First, SPSS 26.0 was used for descriptive statistics, data cleaning, coding, and preprocessing. Second, SmartPLS 4.0 was used to analyze the measurement and structural models. Internal consistency, convergent validity, and discriminant validity were tested sequentially. The effect pathways from digital governance pressure to digital burnout were then examined, and the Bootstrap method was used to test the mediating effects of cognitive load and negative affect. Finally, the built-in NCA module in SmartPLS 4.0 was used to identify the threshold conditions associated with high levels of digital burnout.

## 5 Empirical analysis

### 5.1 PLS-SEM analysis

**Figure 2:**
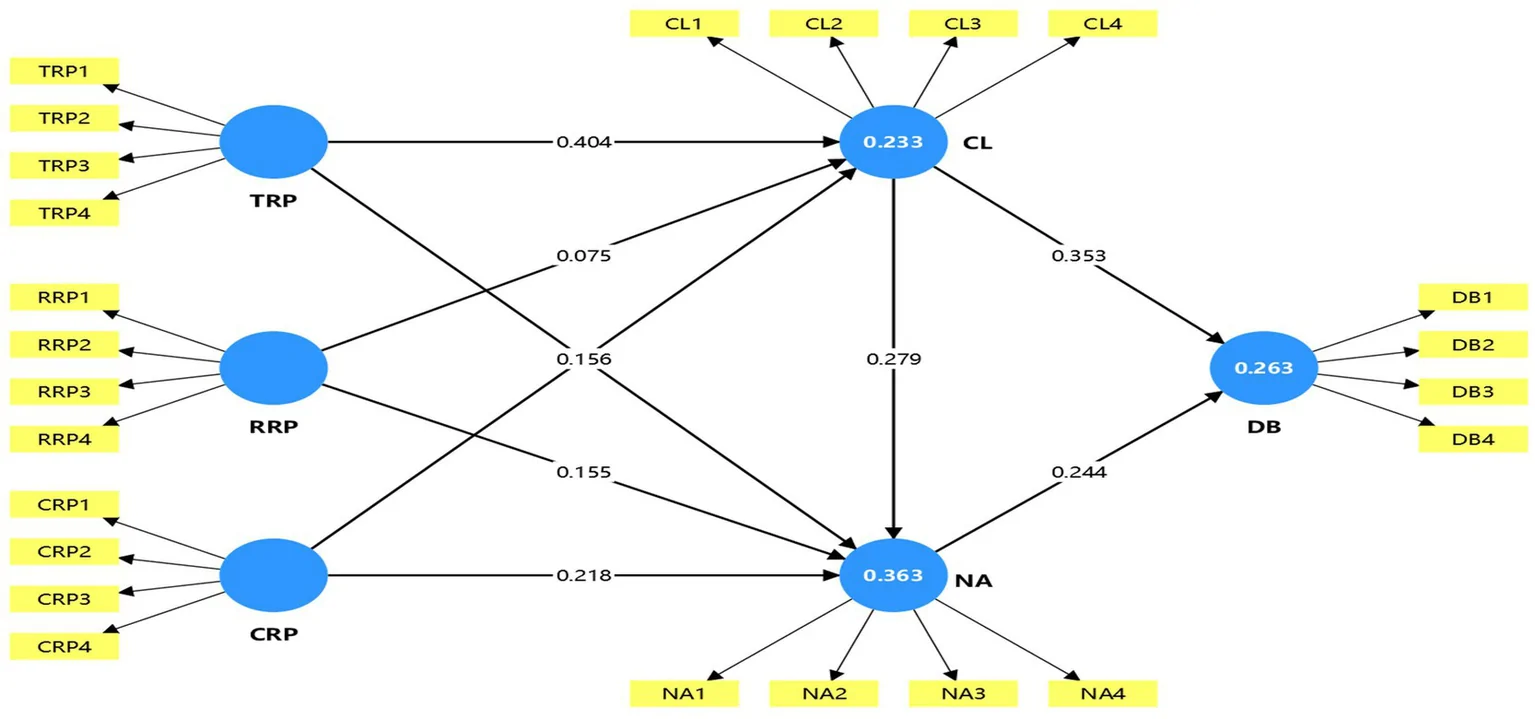
Structural model results.

This study used PLS-SEM to analyze the pathways leading to digital burnout among grassroots public sector employees. The structural equation model results are shown in Figure 2.

#### 5.1.1 Measurement model

First, internal consistency reliability was assessed using composite reliability (CR) and Cronbach’s alpha. Previous studies suggest that CR and Cronbach’s alpha values above 0.70 indicate satisfactory reliability (Hair et al., 2022). As shown in Table 4, all latent constructs had CR and Cronbach’s alpha values above 0.80, indicating good internal consistency. Second, convergent validity was assessed using standardized outer loadings and average variance extracted (AVE). In general, factor loadings above 0.70 and AVE values above 0.50 indicate satisfactory convergent validity. As shown in Table 4, all measurement items had factor loadings above 0.80, and all latent variables had AVE values above 0.70. These results indicate good convergent validity of the measurement scale.

**Table 4:** Measurement model results.

Variables	Items	Factor loadings	Cronbach’s Alpha	CR	AVE
Technology-related pressure	TRP1	0.815	0.873	0.914	0.726
	TRP2	0.870			
	TRP3	0.857			
	TRP4	0.864			
Regulation-related pressure	RRP1	0.866	0.880	0.917	0.735
	RRP2	0.847			
	RRP3	0.842			
	RRP4	0.873			
Context-related pressure	CRP1	0.852	0.875	0.914	0.728
	CRP2	0.860			
	CRP3	0.825			
	CRP4	0.874			
Cognitive load	CL1	0.851	0.869	0.910	0.717
	CL2	0.851			
	CL3	0.860			
	CL4	0.825			
Negative affect	NA1	0.852	0.863	0.907	0.709
	NA2	0.835			
	NA3	0.849			
	NA4	0.832			
Digital burnout	DB1	0.851	0.857	0.903	0.700
	DB2	0.818			
	DB3	0.834			
	DB4	0.844			

Finally, discriminant validity was examined using the Fornell-Larcker criterion and the heterotrait-monotrait ratio (HTMT). According to the Fornell and Larcker (1981) criterion, a latent variable has good discriminant validity when the square root of its AVE is greater than its correlations with other latent variables. As shown in Table 5, the square root of the AVE for each latent variable was greater than its correlations with the other latent variables. The HTMT results also show that all HTMT values among the constructs were below the recommended threshold of 0.85 (Hair et al., 2022). These results indicate satisfactory discriminant validity.

**Table 5:** Discriminant validity results.

Construct	Fornell-Larcker criterion						HTMT ratio (<0.85)					
	TRP	RRP	CRP	CL	NA	DB	TRP	RRP	CRP	CL	NA	DB
TRP	**0.852**											
RRP	0.437	**0.857**					**0.492**					
CRP	0.494	0.475	**0.853**				0.56	**0.534**				
CL	0.471	0.285	0.306	**0.847**			0.539	0.32	**0.348**			
NA	0.463	0.406	0.454	0.463	**0.842**		0.526	0.462	0.520	**0.532**		
DB	0.454	0.457	0.432	0.465	0.407	**0.837**	0.518	0.521	0.494	0.536	**0.468**	

Bold diagonal values in the Fornell-Larcker section represent the square roots of AVE. Off-diagonal values represent inter-construct correlations. All HTMT values are below the recommended threshold of 0.85, indicating satisfactory discriminant validity. TRP, technology-related pressure; RRP, regulation-related pressure; CRP, context-related pressure; CL, cognitive load; NA, negative affect; DB, digital burnout. The same abbreviations are used hereafter.

This study also used the variance inflation factor (VIF) to examine whether multicollinearity existed among the predictor variables. In general, VIF values below 5, or below the more conservative threshold of 3.3, indicate that serious multicollinearity is unlikely (Kock, 2015; Hair et al., 2022). The VIF values of all predictor variables ranged from 1.272 to 1.633, indicating that the structural model did not have a serious multicollinearity problem.

To further examine common method bias, we used Harman’s single-factor test, full collinearity VIF, and confirmatory factor analysis. First, Harman’s single-factor test extracted six factors with eigenvalues greater than 1, and the first common factor explained 37.77% of the total variance, below the 50% threshold. No single factor therefore explained most of the variance, suggesting a relatively low risk of common method bias (Podsakoff et al., 2003; Kock, 2015). Second, the full collinearity VIF test showed that the VIF values of all latent variables ranged from 1.508 to 1.694, below the recommended threshold of 3.3. This further indicates that common method bias was unlikely to seriously affect the main conclusions (Kock, 2015). Finally, we used Mplus 8.3 to conduct confirmatory factor analysis and compared a single-factor CFA model with the theoretical six-factor CFA model. The single-factor CFA model showed poor fit: chi-square/df = 16.674, CFI = 0.725, TLI = 0.699, RMSEA = 0.171, and SRMR = 0.110. By contrast, the six-factor CFA model showed good fit: chi-square/df = 1.632, CFI = 0.990, TLI = 0.988, RMSEA = 0.034, and SRMR = 0.025. These results indicate that the measurement items cannot be adequately explained by one common factor and that the risk of common method bias was low.

#### 5.1.2 Evaluation of the structural model

The quality of the structural model was evaluated using the coefficient of determination (R^2^), adjusted coefficient of determination (Adjusted R^2^), cross-validated redundancy (Q^2^), and the standardized root mean square residual (SRMR). Previous studies suggest that R^2^ can be used to assess the explanatory power of a model for endogenous variables, and that an R^2^ value of 0.10 or above indicates acceptable explanatory power in behavioral and social science research (Falk and Miller, 1992; Chin, 1998; Hair et al., 2022). The R^2^ values for cognitive load, negative affect, and digital burnout were 0.233, 0.363, and 0.263, respectively; the corresponding adjusted R^2^ values were 0.229, 0.359, and 0.260. These results indicate that the model has acceptable explanatory power for each endogenous variable. In addition, Q^2^ was used to assess predictive relevance. In general, a Q^2^ value greater than 0 indicates predictive relevance (Geisser, 1974; Hair et al., 2022). The Q^2^ values for cognitive load, negative affect, and digital burnout were 0.165, 0.252, and 0.180, respectively, indicating good predictive relevance for each endogenous variable. Furthermore, SRMR was used as an auxiliary indicator of model fit. Previous studies generally regard an SRMR value below 0.10 as acceptable and a value below 0.08 as relatively good (Henseler et al., 2014; Hair et al., 2022). The SRMR value of the model was 0.077, below the recommended threshold of 0.08. Table 6 reports the R^2^, adjusted R^2^, Q^2^, and SRMR results of the structural model.

**Table 6:** Quality of the structural model.

Endogenous construct	R^2^	Adjusted R^2^	Q^2^
CL	0.233	0.229	0.165
NA	0.363	0.359	0.252
DB	0.263	0.26	0.18
Model fit index	Value		
SRMR	0.077		

R^2^ = coefficient of determination; Q^2^ = Stone-Geisser’s predictive relevance; SRMR values below 0.08 indicate acceptable model fit.

After the quality of the structural model was confirmed, the bootstrap method was used to test the significance of the hypothesized paths. The number of bootstrap samples was set at 5,000. The significance of each path was evaluated based on the standardized path coefficient (*β*), t value, *p* value, and effect size (f^2^). Table 7 reports the path analysis results.

**Table 7:** Results of path effects and hypothesis testing.

Path	*β*	*t*	*p*	f^2^	Result
TRP → CL	0.404	9.071	***	0.15	Supported
TRP → NA	0.156	3.176	**	0.023	Supported
RRP → CL	0.075	1.632	0.103	0.005	Not supported
RRP → NA	0.155	3.562	***	0.027	Supported
CRP → CL	0.071	1.494	0.135	0.004	Not supported
CRP → NA	0.218	4.59	***	0.05	Supported
CL → NA	0.279	6.32	***	0.094	Supported
CL → DB	0.353	7.58	***	0.132	Supported
NA → DB	0.244	4.87	***	0.063	Supported
TRP → CL → DB	0.142	5.557	***	——	Supported
RRP → CL → DB	0.026	1.491	0.136	——	Not supported
CRP → CL → DB	0.025	1.442	0.149	——	Not supported
TRP → NA → DB	0.038	2.489	*	——	Supported
RRP → NA → DB	0.038	2.549	*	——	Supported
CRP → NA → DB	0.053	3.073	**	——	Supported
TRP → CL → NA → DB	0.027	3.702	***	——	Supported
RRP → CL → NA → DB	0.005	1.527	0.127	——	Not supported
CRP → CL → NA → DB	0.005	1.363	0.173	——	Not supported

***p < 0.001, **p < 0.01, *p < 0.05.

For the direct path effects, technology-related pressure was significantly and positively associated with cognitive load (*β* = 0.404, *t* = 9.071, *p* < 0.001), with an effect size close to the medium level (f^2^ = 0.150). This indicates that technology-related pressure is an important antecedent of cognitive load among grassroots public sector employees. Technology-related pressure was also significantly and positively associated with negative affect (*β* = 0.156, *t* = 3.176, *p* < 0.01), although the effect size was small (f^2^ = 0.023). Therefore, H1a and H1b were supported. By contrast, the relationship between regulation-related pressure and cognitive load was not significant (*β* = 0.075, *t* = 1.632, *p* = 0.103), and the relationship between context-related pressure and cognitive load was also not significant (*β* = 0.071, *t* = 1.494, *p* = 0.135). Their effect sizes were only 0.005 and 0.004, respectively, indicating weak explanatory effects on cognitive load. Therefore, H2a and H3a were not supported. However, regulation-related pressure was significantly and positively associated with negative affect (*β* = 0.155, *t* = 3.562, *p* < 0.001), and context-related pressure was also significantly and positively associated with negative affect (*β* = 0.218, *t* = 4.590, *p* < 0.001). Their effect sizes were 0.027 and 0.050, respectively, indicating statistically significant but small effects. These results suggest that regulation-related and context-related pressure do not mainly operate through cognitive load but are more clearly reflected in emotional threat effects. Therefore, H2b and H3b were supported. In addition, cognitive load was significantly and positively associated with negative affect (*β* = 0.279, *t* = 6.320, *p* < 0.001), indicating that higher cognitive load is usually accompanied by stronger negative affect. Thus, H4 was supported. Cognitive load was also significantly and positively associated with digital burnout (*β* = 0.353, *t* = 7.580, *p* < 0.001), with an effect size close to the medium level (f^2^ = 0.132). Negative affect was likewise significantly and positively associated with digital burnout (*β* = 0.244, *t* = 4.870, *p* < 0.001), with a small effect size (f^2^ = 0.063). Therefore, H5 and H6 were supported.

For the mediation effects, the indirect effect of technology-related pressure on digital burnout through cognitive load was significant (*β* = 0.142, *t* = 5.557, *p* < 0.001), indicating that cognitive load mediates the relationship between technology-related pressure and digital burnout. Therefore, H7a was supported. However, the indirect effect of regulation-related pressure on digital burnout through cognitive load was not significant (*β* = 0.026, *t* = 1.491, *p* = 0.136), so H7b was not supported. Similarly, the indirect effect of context-related pressure on digital burnout through cognitive load was not significant (*β* = 0.025, *t* = 1.442, *p* = 0.149), so H7c was not supported. For the simple mediating role of negative affect, the indirect effect of technology-related pressure on digital burnout through negative affect was significant (*β* = 0.038, *t* = 2.489, *p* < 0.05). The indirect effect of regulation-related pressure on digital burnout through negative affect was also significant (*β* = 0.038, *t* = 2.549, *p* < 0.05), as was the indirect effect of context-related pressure on digital burnout through negative affect (*β* = 0.053, *t* = 3.073, *p* < 0.01). Therefore, H8a, H8b, and H8c were supported. For the serial mediation paths, technology-related pressure had a significant indirect association with digital burnout through the sequential path of cognitive load to negative affect (*β* = 0.027, *t* = 3.702, *p* < 0.001). Thus, H9a was supported. By contrast, the chain-mediating effects of regulation-related pressure and context-related pressure were not significant. The corresponding results were *β* = 0.005, *t* = 1.527, *p* = 0.127 and *β* = 0.005, *t* = 1.363, *p* = 0.173, respectively. Therefore, H9b and H9c were not supported.

### 5.2 Necessary condition analysis (NCA)

To further identify the threshold patterns associated with high levels of digital burnout, we introduced necessary condition analysis (NCA) after the sufficiency-based PLS-SEM analysis. Unlike PLS-SEM, which mainly reveals net-effect relationships among variables, NCA is used to examine whether an outcome can reach a high level only when a condition reaches a certain minimum level. The built-in NCA function in SmartPLS 4.0 was used to examine whether technology-related pressure, regulation-related pressure, context-related pressure, cognitive load, and negative affect show necessary-condition patterns in relation to digital burnout.

#### 5.2.1 Preliminary observation of necessary condition characteristics

**Figure 3:**
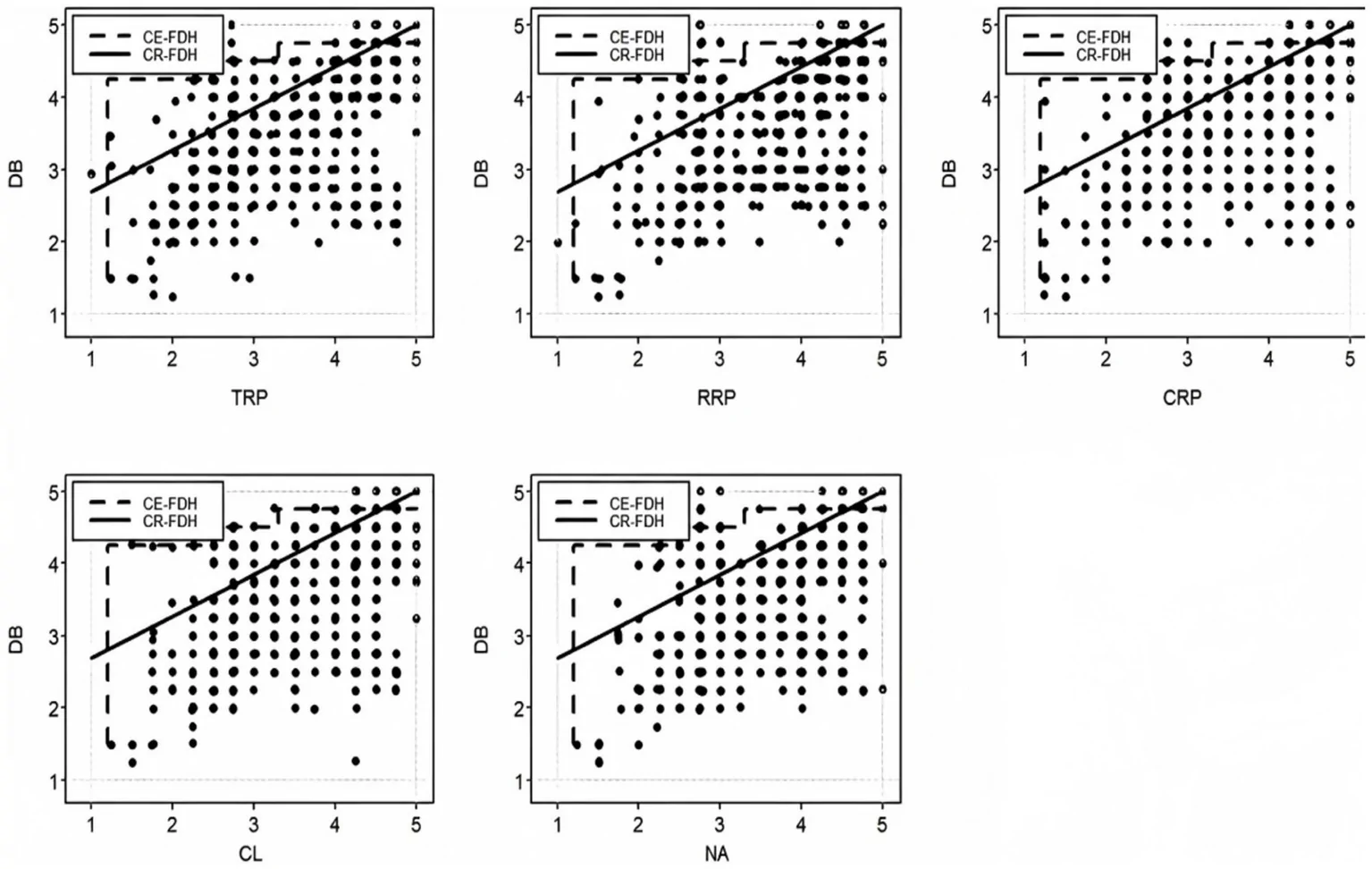
NCA scatter plot analysis.

Figure 3 presents the NCA scatter plots of each antecedent variable relative to digital burnout. In general, an empty area in the upper-left corner of a scatter plot suggests a possible necessary-condition pattern: high outcome values are not observed when the condition variable is below a certain level. As shown in Figure 3, the scatter plots for technology-related pressure, regulation-related pressure, context-related pressure, cognitive load, and negative affect all show upper-left empty areas to varying degrees. These results indicate that these variables may impose minimum-level constraints on high levels of digital burnout. Whether these necessity patterns are statistically significant is further assessed using the effect size d and the permutation test.

#### 5.2.2 Testing of necessary condition effects

To determine whether the observed necessary-condition patterns are statistically significant, we applied the CE-FDH method to calculate the effect size d and the *p* value from the permutation test. In NCA, the effect size d measures the extent to which a condition constrains the outcome, while the permutation test assesses whether the observed necessary-condition effect is statistically significant. This reduces the risk of falsely identifying a necessary-condition effect (Dul, 2016; Dul et al., 2020). When *p* < 0.05, the condition variable is considered to show a significant necessary-condition effect on the outcome; when *p* > 0.05, the variable does not constitute a significant necessary condition. As shown in Table 8, the necessary-condition effects of technology-related pressure (TRP), regulation-related pressure (RRP), context-related pressure (CRP), cognitive load (CL), and negative affect (NA) on digital burnout were all statistically significant. According to the criteria for interpreting NCA effect sizes, 0 < d < 0.1 indicates a low-level effect, 0.1 < = d < 0.5 indicates a medium-level effect, and d > = 0.5 indicates a high-level effect (Dul, 2016). The effect size of technology-related pressure was 0.095, indicating a low-level necessary-condition effect. The effect sizes of regulation-related pressure, context-related pressure, cognitive load, and negative affect were 0.122, 0.119, 0.199, and 0.217, respectively, indicating medium-level necessary-condition effects.

**Table 8:** Results of necessary condition analysis (NCA).

Antecedent condition	Accuracy (%)	Ceiling zone	d	*p*	Necessity level
TRP	100	1.757	0.095	**	Low necessity
RRP	100	1.502	0.122	***	Medium necessity
CRP	100	1.248	0.119	***	Medium necessity
CL	100	1.489	0.199	***	Medium necessity
NA	100	2.238	0.217	***	Medium necessity

TRP, technology-related pressure; RRP, regulation-related pressure; CRP, context-related pressure; CL, cognitive load; NA, negative affect; DB, digital burnout. ***p < 0.001, **p < 0.01.

#### 5.2.3 Bottleneck level analysis

Table 9 reports the minimum bottleneck values required for each variable at different levels of digital burnout. The bottleneck values identified by NCA should not be interpreted as causal trigger points; reaching a given value does not mean that digital burnout will necessarily occur. Instead, the values indicate that, in this sample, when digital burnout reaches a corresponding level, the relevant condition is usually not observed below the indicated minimum level. Overall, as the level of digital burnout increases, the minimum requirements for the variables generally rise. This suggests that higher levels of digital burnout are rarely observed when only one factor remains at a low level. Instead, multiple conditions tend to reach at least certain minimum levels. For example, when digital burnout reaches the 70% level, the required minimum values are 1.502 for technology-related pressure, 1.248 for regulation-related pressure, 1.757 for context-related pressure, 1.489 for cognitive load, and 2.000 for negative affect. These values indicate lower-bound reference points rather than sufficient causes of burnout. Combined with Table 9, the results also show that the minimum bottleneck values of cognitive load and negative affect are relatively higher than those of the three external pressure variables. These results indicate that cognitive resource depletion and the accumulation of negative affect have more evident threshold patterns in the formation of high levels of digital burnout, especially at higher outcome levels.

**Table 9:** Bottleneck table for digital burnout.

Digital burnout level (%)	TRP	RRP	CRP	CL	NA
0%	—	1.244	—	1.246	1.238
10%	—	1.244	—	1.246	1.238
20%	—	1.244	—	1.489	1.733
30%	1.220	1.248	—	1.489	1.733
40%	1.498	1.248	—	1.489	1.733
50%	1.502	1.248	1.235	1.489	1.733
60%	1.502	1.248	1.235	1.489	1.733
70%	1.502	1.248	1.757	1.489	2.000
80%	2.000	2.000	1.757	1.489	2.238
90%	2.000	2.000	1.757	3.245	2.248
100%	2.000	2.500	2.721	4.243	2.753

TRP, technology-related pressure; RRP, regulation-related pressure; CRP, context-related pressure; CL, cognitive load; NA, negative affect; DB, digital burnout.

## 6 Discussion

### 6.1 Research findings

Our research is situated in rural digital governance in China. Based on the SOR model, it examines how technology-related pressure, regulation-related pressure, and context-related pressure are associated with digital burnout among grassroots public sector employees through cognitive load and negative affect. It addresses the question of whether pressures from different sources operate through homogeneous psychological mechanisms when digital technology is deeply embedded in grassroots governance. The results show that digital governance pressure does not affect grassroots public sector employees in a homogeneous way. Instead, different pressure sources are associated with digital burnout through distinct psychological pathways.

(1) Technology-related pressure is positively associated with both cognitive load and negative affect. This result aligns with technostress theory. The complexity, uncertainty, and frequent updating of digital technology may increase individuals’ information-processing pressure and adaptation burden (Consiglio et al., 2023; Bondanini et al., 2020). The findings also support Kumar’s (2024) argument that continuous technology learning and frequent system updates may trigger technology-related anxiety. In grassroots digital governance, technology-related pressure is not only a source of cognitive depletion but also an affective threat. Complex platform operations, frequent system updates, and technology adaptation requirements may increase the information-processing burden of grassroots public sector employees and trigger negative emotions such as tension, anxiety, and helplessness. Through cognitive load and negative affect, these pressures may further increase the risk of digital burnout.

(2) Regulation-related pressure and context-related pressure are positively associated with negative affect, but their associations with cognitive load are not significant. This evidence contributes to studies on administrative burden, digital red tape, and street-level bureaucracy. Existing studies suggest that the interaction between digital technology and bureaucratic systems may create new digital compliance costs and digital red tape through online reporting, digital trace-making, format requirements, and performance evaluation (Peeters, 2023; Liu et al., 2025). Street-level bureaucrats also need to repeatedly explain, coordinate, and translate between system requirements, organizational tasks, and citizens’ needs during policy implementation (Lipsky, 1980; Ding, 2022; Dai et al., 2026). Theoretically, these processes may increase the pressure of rule identification, procedural compliance, and information verification (Moynihan et al., 2015; Peeters, 2023). However, the results of this study show that regulation-related and context-related pressure do not appear primarily as cognitive depletion pressure in grassroots digital governance. Instead, they are more clearly reflected as affective threat pressure. For regulation-related pressure, tasks such as online reporting, format requirements, and digital trace-making have strong procedural features. Their core does not always lie in complex information processing; rather, they often involve repeated compliance with fixed procedures, format requirements, and regulatory rules (Heggertveit et al., 2022; Liu et al., 2025). After long-term adaptation, grassroots public sector employees may not necessarily perceive these tasks as highly complex information-processing burdens. However, time limits, process tracking, and responsibility-tracing mechanisms can easily trigger tension, anxiety, and helplessness. For context-related pressure, affective threat mainly comes from the tension between standardized digital rules and complex grassroots realities. Grassroots governance tasks are often highly contextual and non-standardized. When real-world problems cannot be fully absorbed by standardized platform procedures, grassroots public sector employees must constantly explain, coordinate, and translate between system rules, organizational tasks, and citizens’ needs (Ding, 2022; Dai et al., 2026). Such pressure may not appear as a stable information-processing burden, but it can lead to difficulties in rule adaptation, coordination pressure, and emotional frustration. Overall, regulation-related and context-related pressure come from different sources, but both may increase digital burnout risk through the affective threat pathway. This finding does not clearly support the view that compliance costs and contextual pressure increase cognitive load (Moynihan et al., 2015; Peeters, 2023; Li and Pang, 2026). However, it supports the argument in administrative burden and street-level bureaucracy research that institutional requirements, accountability pressure, and reduced discretion may trigger psychological stress (Hattke et al., 2020; Overman et al., 2025; Herd et al., 2023).

(3) Cognitive load and negative affect are both positively associated with digital burnout, and cognitive load is further positively associated with negative affect. This result supports the explanatory logic of the SOR model and the cognitive-affective pathway. The SOR model suggests that external stimuli do not directly translate into individual responses but must be processed through internal cognitive and affective states (Mehrabian and Russell, 1974). In this study, digital burnout among grassroots public sector employees is not only related to external digital governance pressure. It is also closely linked to the depletion of cognitive and emotional resources. The mediation results further show that technology-related pressure is indirectly associated with digital burnout through cognitive load, negative affect, and the sequential pathway from cognitive load to negative affect. By contrast, regulation-related and context-related pressure are mainly indirectly associated with digital burnout through negative affect. These results indicate that digital burnout is not simply the direct result of external digital governance pressure. Rather, it is an occupational health risk that gradually accumulates after external pressure is processed through individuals’ internal cognitive processing and affective responses. This finding refines the explanatory pathway of the SOR model in the context of grassroots digital governance. It advances the broad understanding of how digital pressure operates to a more nuanced explanatory level of “different digital governance pressures–differentiated psychological processing–digital burnout.” In doing so, it addresses the limitations of previous studies on digital pressure and digital burnout.

(4) The NCA results suggest that high levels of digital burnout are not associated with a single pressure source alone. Instead, they tend to appear when multiple antecedent conditions reach certain minimum levels. Compared with the three forms of external digital governance pressure, cognitive load and negative affect show stronger necessary-condition patterns in relation to digital burnout. These results indicate that cognitive resource depletion and accumulated negative emotions may function as more salient psychological threshold conditions in the formation of high levels of digital burnout. This pattern should be understood as identifying lower-bound conditions rather than deterministic causal triggers. It enriches the explanation of the psychological mechanisms through which digital governance pressure is associated with digital burnout and extends digital burnout research in grassroots digital governance.

### 6.2 Theoretical and methodological contributions

(1) This study extends digital burnout research to rural digital governance in China and enriches research on the unintended consequences of digital transformation in the public sector. Existing studies on digital burnout have mainly focused on general technology use, private-sector work settings, education, and healthcare (Erten and Özdemir, 2020; Islam et al., 2020; Wang et al., 2026). Relatively limited attention has been paid to digital burnout in the public sector, especially among grassroots public sector employees. The findings show that digital burnout among grassroots public sector employees is not simply caused by more frequent technology use. it represents a complex occupational health risk formed through the interaction of technological rules, institutional requirements, and grassroots governance realities (Moynihan et al., 2015; Liu et al., 2025). From this perspective, the study extends digital burnout research from general technology-use contexts to public governance implementation and provides new empirical evidence for understanding the potential negative effects of digital public governance.

(2) This study enriches the application of the SOR model in public sector digital governance research. The SOR model emphasizes that external stimuli are processed through internal organismic states before being associated with individual responses (Mehrabian and Russell, 1974). Existing SOR studies have mainly explained consumer behavior, digital platform use, online service experience, and general organizational behavior. Its application to grassroots public sector digital governance remains limited.

This study defines digital governance pressure as the external stimulus, cognitive load and negative affect as organismic states, and digital burnout as the individual response. Following this logic, it constructs an analytical framework of digital governance pressure-cognitive and affective processing-digital burnout. In doing so, the study confirms the explanatory power of the SOR model in grassroots digital governance and shows that public sector digital transformation does not act directly on grassroots public sector employees. Instead, its effects are processed through cognitive resource depletion and affective responses.

(3) This study reveals the differentiated psychological mechanisms through which different forms of digital governance pressure are transformed into digital burnout. Existing studies have examined technological pressure (Fleischer and Wanckel, 2024), administrative burden (George et al., 2021; Johnson and Kroll, 2023), and street-level bureaucratic dilemmas (Busch and Zinner Henriksen, 2018) caused by public sector digital transformation from technological, institutional, and actor-oriented perspectives. These studies provide an important foundation for understanding digital burnout among public sector employees. However, most adopt a single theoretical perspective and provide limited explanation of how different pressure sources are transformed into digital burnout through differentiated psychological pathways. By integrating technological, institutional, and actor-oriented perspectives, we conceptualize the external stimuli perceived by grassroots public sector employees as technology-related pressure, regulation-related pressure, and context-related pressure. It further explains the internal pathways through which these pressures are associated with digital burnout via cognitive load and negative affect. The findings show that technology-related pressure mainly produces cognitive depletion, accompanied by some emotional activation, whereas regulation-related and context-related pressure are mainly characterized by emotional threat. In terms of effect pathways, technology-related pressure is associated with digital burnout through the independent mediation of cognitive load and the chain mediation of cognitive load to negative affect. In contrast, regulation-related and context-related pressure operate primarily through the negative affect pathway. In this way, the present work moves existing research from asking whether digital pressure is associated with digital burnout to explaining how different forms of digital governance pressure are associated with digital burnout through different psychological mechanisms.

(4) This study combines PLS-SEM and NCA to deepen the understanding of digital burnout from both sufficient-pathway and necessary-condition perspectives. The PLS-SEM results reveal pathways through which different forms of digital governance pressure are associated with digital burnout. The NCA results further identify minimum threshold patterns associated with high levels of digital burnout. In this way, the study not only answers which factors are associated with digital burnout but also identifies which conditions need to reach certain lower-bound levels when high digital burnout is observed. This provides a more complete analytical perspective for understanding the formation mechanism of digital burnout among grassroots public sector employees.

### 6.3 Practical implications

Our findings carry implications for grassroots digital governance and occupational health in the public sector.

First, grassroots digital governance should shift from platform expansion to platform burden reduction to reduce technology-related pressure among grassroots public sector employees. Existing studies show that digital government does not necessarily reduce administrative burden. When digital systems lack integration, they may create new digital administrative burdens through multiple platform logins, repeated data entry, data verification, and procedural operations (Heggertveit et al., 2022; Peeters, 2023). This study finds that technology-related pressure is significantly associated with cognitive load, suggesting that adaptation to digital applications, systems, and governance platforms is an important source of digital burnout. Therefore, reducing grassroots burdens should not only mean reducing the number of tasks. It should also reduce the cognitive burden caused by the parallel use of multiple platforms, repeated reporting, frequent system updates, and complex operating rules (Liang et al., 2024).

Second, digital performance evaluation should be optimized to reduce regulation-related pressure and accountability-related emotional burden. Digital assessment can improve the standardization and traceability of governance processes. However, if it relies excessively on formal indicators such as check-in frequency, digital trace records, and ranking results, grassroots public sector employees may spend much of their energy meeting platform requirements rather than solving real governance problems (Dai et al., 2026). Existing studies also show that excessive reliance on performance measurement and administrative control in grassroots governance may weaken the responsiveness and professional judgment of frontline workers (Lewis, 2015). Our results further show that regulation-related pressure is associated with digital burnout through negative affect. Excessive emphasis on online trace-making, real-time ranking, process monitoring, and responsibility tracing may therefore keep grassroots public sector employees in a long-term psychological state of being monitored and evaluated, which is not conducive to the healthy operation of grassroots governance (Miao et al., 2025).

Accordingly, the content and methods of digital performance evaluation should be optimized. Formalistic tendencies in evaluation should be avoided, and evaluation indicators should be more adaptable to grassroots contexts, more sensitive to local conditions, and more outcome oriented. The focus of evaluation should shift from whether platform traces are complete to whether governance problems have been effectively solved.

Third, contextualized discretionary space in grassroots governance should be preserved to avoid excessive compression of governance flexibility by standardized digital rules. Studies on street-level bureaucracy and digital government show that digital tools may reshape the discretionary space of frontline workers by compressing, empowering, or reproducing street-level decision-making, depending on the context (Buffat, 2015; Marienfeldt, 2024). This study finds that context-related pressure is mainly associated with digital burnout through negative affect. Grassroots public sector employees may therefore experience emotional frustration and helplessness when they explain, coordinate, and translate between standardized digital systems and complex grassroots realities. Digital platform design and governance assessment should not require them to mechanically transform all governance processes into standardized data. Instead, they should allow room for interpretation and contextualized discretion in special cases, complex affairs, and public consultation.

Fourth, a risk-warning and organizational support mechanism for digital burnout should be established. The findings show that cognitive load and negative affect play important roles in the formation of digital burnout. The NCA results also suggest that these two factors have clear threshold significance in identifying the risk of high digital burnout. Organizational managers should therefore focus not only on platform operation efficiency but also on the psychological burden experienced by grassroots public sector employees in digital governance. Targeted technical training, peer support, psychological support, and workflow optimization should be provided to avoid transferring the pressure of digital governance entirely to individual employees. The goal of grassroots digital governance should not only be to make data more complete and processes more visible. More importantly, digital tools should support grassroots public sector employees in carrying out governance work more effectively and sustainably.

### 6.4 Limitations and future research

This study has several limitations. First, it uses cross-sectional survey data, which makes it difficult to fully reveal the dynamic relationships among digital governance pressure, cognitive load, negative affect, and digital burnout. Future studies could use longitudinal data or follow-up research designs to further examine the causal mechanisms among these variables. In addition, we relied on convenience sampling and snowball sampling. Although this approach helped reach geographically dispersed grassroots public sector employees with practical experience in digital governance, it may lead to sample selection bias. Future research could combine stratified sampling, multi-region probability sampling, or administrative data matching to improve the robustness and external validity of the findings.

Second, although our approach combines PLS-SEM and NCA to reveal the formation mechanism of digital burnout among grassroots public sector employees from sufficient-pathway and necessary-condition perspectives, the current analysis mainly identifies variable relationships at the overall sample level. It does not fully examine possible heterogeneity in digital burnout across individuals. Future research could use person-centered methods, such as latent profile analysis (LPA), to identify different types of digital burnout among grassroots public sector employees. Qualitative comparative analysis (QCA) could also be used to explore how digital governance pressures, cognitive load, and negative affect combine as complex conditions associated with high levels or different types of digital burnout. Third, our analysis focuses on rural digital governance in China. The applicability of its findings to different regions, administrative levels, and governance contexts in other countries requires further examination. Future studies could incorporate variables such as digital literacy, organizational support, platform integration, and institutional environment into the analytical framework to further extend understanding of the formation mechanism of digital burnout among grassroots public sector employees.

## Data Availability

The datasets generated and/or analyzed during the current study are not publicly available because of privacy and ethical restrictions related to preserving the anonymity of grassroots public sector employees, but they are available from the corresponding author on reasonable request.
